# Acceptability of multimodal pelvic floor physical therapy to treat dyspareunia after gynecological malignancies: a qualitative study of women’s views and experiences

**DOI:** 10.1007/s00192-022-05304-4

**Published:** 2022-08-10

**Authors:** Marie-Pierre Cyr, Rosalie Dostie, Chantal Camden, Chantale Dumoulin, Paul Bessette, Annick Pina, Walter Henry Gotlieb, Korine Lapointe-Milot, Marie-Hélène Mayrand, Mélanie Morin

**Affiliations:** 1 School of Rehabilitation, Faculty of Medicine and Health Sciences, University of Sherbrooke, 3001 12e Avenue N, Sherbrooke, Quebec J1H 5N4 Canada; 2 Research Center of the Centre Hospitalier Universitaire de Sherbrooke, 3001 12e Avenue N, Sherbrooke, Quebec J1H 5H3 Canada; 3 School of Health and Rehabilitation Sciences, Faculty of Health and Behavioural Sciences, The University of Queensland, 84a Services Road, St Lucia, Brisbane, Queensland 4072 Australia; 4 School of Rehabilitation, Faculty of Medicine, University of Montreal, 7077 Park Avenue, Montreal, Quebec H3N 1X7 Canada; 5 Research Center of the Institut Universitaire de Gériatrie de Montréal, 4545 Queen Mary, Montreal, Quebec H3W 1W6 Canada; 6 Department of Obstetrics and Gynecology, Division of Gynecologic Oncology, Faculty of Medicine and Health Sciences, University of Sherbrooke, 3001 12e Avenue N, Sherbrooke, Quebec J1H 5N4 Canada; 7 Department of Obstetrics and Gynecology, Division of Gynecologic Oncology, Faculty of Medicine, University of Montreal, PO Box 6128, Centre-ville Station, Montreal, Quebec H3C 3J7 Canada; 8 Research Center of the Centre Hospitalier de l’Université de Montréal, 900 St Denis St, Montreal, Quebec H2X 0A9 Canada; 9 Department of Obstetrics and Gynecology, Division of Gynecologic Oncology, Faculty of Medicine, McGill University, 1001 Decarie Blvd, Montreal, Quebec H4A 3J1 Canada; 10 Lady Davis Institute of the Jewish General Hospital, 3755 Chemin de la Côte-Sainte-Catherine, Montreal, Quebec H3T 1E2 Canada; 11 Departments of Obstetrics and Gynecology and Social and Preventive Medicine, Faculty of Medicine, University of Montreal, PO Box 6128, Centre-ville Station, Montreal, Quebec H3C 3J7 Canada

**Keywords:** Behavior mechanisms, Cancer survivors, Pain, Physical therapy, Women’s health

## Abstract

**Introduction and hypothesis:**

Multimodal pelvic floor physical therapy (PFPT) is recommended after gynecological malignancies to treat dyspareunia. However, data to strongly support its implementation in the cancer care continuum are lacking. The aim of this study was to explore the views and experiences of gynecological cancer survivors with dyspareunia regarding the acceptability of multimodal PFPT.

**Methods:**

This qualitative study was conducted with the participants (*n* = 28) of a study investigating a 12-week multimodal PFPT treatment. Individual semi-structured telephone interviews served to collect qualitative data pertaining to women’s views and experiences of the treatment they received. Interviews were recorded and transcribed for analysis using the interpretative description framework.

**Results:**

Our cohort described the appropriateness of the treatment in terms of modalities, physical therapist, care delivery, and intensity (Theme 1). While the intensity was reported as demanding by a few, all participants stressed that it was relevant to see significant improvements (Theme 2). In addition to the treatment characteristics and women’s beliefs and attitudes, noticing the treatment effects motivated their participation (Theme 2). Women expressed being highly satisfied with the treatment based on their positive experiences and the balance between their efforts and the results they obtained (Theme 3). As a result, they all recommended this treatment (Theme 3).

**Conclusions:**

This is the first study to examine the acceptability of multimodal PFPT in the context of gynecological malignancies. This treatment was found acceptable and can be offered to gynecological cancer survivors.

**Supplementary Information:**

The online version contains supplementary material available at 10.1007/s00192-022-05304-4.

## Introduction

There have been tremendous advances to increase the survival rates of women diagnosed with gynecological malignancies [1], leading to calls for a greater focus on survivorship care. This population is at high risk of developing sexual dysfunctions [2]. Painful sexual intercourse, or dyspareunia, is frequent, afflicting up to 67% of gynecological cancer survivors [3]. Women suffer from psychological distress and relationship issues, which undermine their quality of life [4]. Side effects of cancer treatments such as dyspareunia also tend to persist or worsen over time [5], and women have persistent unaddressed sexual difficulties [5–7].

Pelvic floor physical therapy (PFPT) has been proposed in survivorship guidelines to address dyspareunia [8–10]. This multimodal treatment may entail an educational module, manual therapy techniques, pelvic floor muscle exercises with biofeedback, and home exercises including insertion exercises with a dilator. A recent study has investigated a 12-week multimodal PFPT treatment in a cohort of gynecological cancer survivors with dyspareunia [11]. Results suggested a reduction in pain as well as an improvement in sexual function [11], pelvic floor muscle function [12], and psychosexual outcomes [13] immediately at post-treatment. Data collected at 12-month follow-up suggested that these improvements were maintained over time [14]. Overall, findings indicate that women with dyspareunia could benefit from multimodal PFPT. The evidence further suggests that multimodal PFPT should be considered in the cancer care continuum. However, data informing us whether it could be implemented in clinical settings are scarce.

Acceptability has become a key component in the development and implementation phases of complex treatments (e.g., multimodal treatment) in survivorship care [15, 16]. This multifaceted construct reflects the extent to which people consider a treatment avenue to be appropriate [17]. Appropriateness of treatment is based on cognitive and emotional responses of patients, which have been hypothesized to relate to satisfaction and participation behavior [17, 18]. For instance, if some aspects of a treatment are viewed as inappropriate, patients may not fully participate and may be dissatisfied, questioning whether the treatment could, or even should, be implemented. Accordingly, examining treatment acceptability would provide insight for implementation purposes and help avoid resource waste. To date, only quantitative data concerning the acceptability of multimodal PFPT in gynecological cancer survivors with dyspareunia are available. A mean adherence to home exercises of 88%, a mean attendance rate at treatment sessions of 93%, and an average satisfaction rate of 93% have been reported [11], providing an incomplete perspective of this treatment’s acceptability. Careful consideration of patients’ views and experiences provides the best opportunity to deepen our understanding of treatment acceptability, and their suggestions for improvements can be used to optimize treatment in clinical settings [17, 19].

Given that the development and implementation of effective treatments is a priority to help gynecological cancer survivors preserve or achieve a healthy sexual life [5–7], the aim of this qualitative study was to explore the views and experiences of gynecological cancer survivors with dyspareunia regarding the acceptability of multimodal PFPT treatment.

## Materials and methods

### Study design

This qualitative study was conducted in the Province of Quebec, Canada, and it followed a multicenter prospective interventional study investigating a multimodal PFPT treatment for gynecological cancer survivors with dyspareunia [11–14]. Individual semi-structured telephone interviews were carried out at 12-month follow-up, allowing participants to take a step back from the treatment. The study was approved by the institutional ethics committee, and the interventional study was registered on ClinicalTrials.gov (NCT03935698). Written informed consent was obtained from eligible women agreeing to participate.

### Participants

Thirty-one women who received a diagnosis of endometrial or cervical cancer (stages I-IV) and had completed all cancer treatments for at least 3 months were recruited in the multicenter prospective interventional study. Gynecological cancer survivors had to have suffered regularly from moderate to severe vulvovaginal pain during sexual intercourse for at least 3 months. They also had to have a regular sexual partner and be willing to resume sexual activities with vaginal penetration. The main exclusion criteria were: (1) dyspareunia prior to cancer or pelvic pain unrelated to intercourse, (2) other pelvic conditions (e.g., urinary tract or vaginal infection, deep pelvic pain, chronic constipation, or severe pelvic organ descent) or pelvic surgery unrelated to cancer, (3) other primary pelvic cancer or breast cancer, (4) received PFPT in the last year, and (5) any coexisting significant medical conditions that were likely to interfere with the study procedures.

### Treatment

The treatment was free of charge and consisted of 12 weekly individual sessions of multimodal PFPT that were delivered at a research center facility. Women were under the care of an experienced female physical therapist in pelvic health. Each week, the participants were invited to attend a 60-min in-person session in which the therapist used different modalities to reduce dyspareunia. Information on dyspareunia such as its pathophysiology and how the treatment may help in reducing the pain was provided. The physical therapist gave tips to alleviate and better manage dyspareunia, for instance by using vaginal lubricants, moisturizers, and relaxation and breathing techniques. Women were guided into resuming non-painful sexual activities with their partner. The latter was invited to take part in treatment to learn how to assist their partner in this process. Moreover, the physical therapist gave extensive explanations on how to prevent and treat pelvic floor disorders. Beside all the psychosexual-educational content that was given on hard copy and discussed with the therapist at each session, manual therapy techniques were performed externally and intravaginally on the pelvic floor muscles by the physical therapist. In addition, electromyography biofeedback with an intravaginal probe was used during the session under the supervision of the therapist. Furthermore, women were asked to complete a home exercise program regularly in which the exercises were similar to those carried out during the session. Home exercises entailed relaxation, coordination, strength, and endurance exercises five times per week as well as auto-insertion and desensitization exercises with a finger or vaginal dilator three times per week. It should be noted that all modalities gradually progressed in intensity (e.g., more pressure applied to stretch the tissues, longer duration of the technique or exercise, and greater dilator size), depending on each woman’s progress. The physical therapist also provided feedback on home exercises by means of a diary that was completed daily by the participants. Further details pertaining to the treatment protocol are provided elsewhere [11].

### Data collection

The individual semi-structured telephone interviews lasted approximately 70 min. Prior to the interview, participants were informed about the topics to be discussed. They were also advised to read the documentation they were given during the study to refresh their memory and reflect on their experience. All interviews were conducted by the first author (MPC) who has an expertise in pelvic health, completed qualitative research training, and helped in designing the treatment but was not involved in the participants’ care. A nonjudgmental approach was used to create a genuine respectful relationship to ease the discussion about what could be perceived by women as intimate topics. The interviews were audio-recorded with the prior consent of the women. The interviewer used open-ended questions as well as probing questions addressing the following: (1) the women’s views and experiences of multimodal PFPT regarding its appropriateness, (2) the women’s participation, and (3) the women’s degree of satisfaction and suggestions for improvements. The interviews followed a semi-structured guide (Supplementary material), intersecting with the framework proposed by Sekhon et al. [17]. In addition, participants were asked if there were any changes in their health and if they had sought or undergone other treatments for dyspareunia or sexual dysfunction in the last 12 months.

### Sample size

All women who participated in the treatment were invited to take part in the individual semi-structured telephone interview, regardless of their treatment response, to provide various views and experiences.

### Data analysis

The first author (MPC) performed verbatim transcription of each interview and analyzed the transcripts using NVivo (version 12) software. To ensure data-driven analyses and interpretations, an inductive approach was adopted where the first author (MPC) applied codes to key ideas and then identified emerging themes [20]. Subsequently, the codes were reviewed (RD followed by MM and CC), and coding disagreements were discussed until a consensus was reached. Several meetings were convened to regroup codes into themes and subthemes. Relationships between themes and subthemes were explored by observing patterns across them. Field notes were used to explore researcher reflexivity and further support the interpretation of data. Quotations in English (*n* = 2) and quotations freely translated from French to English and revised by a certified translator (*n* = 26) were selected to illustrate the women’s input.

## Results

**Fig. 1:**
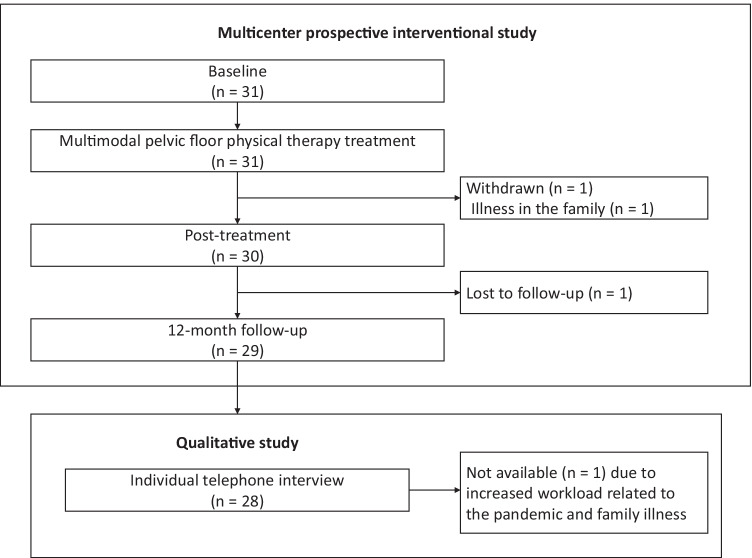
Flowchart

Of the 31 gynecological cancer survivors with dyspareunia who participated in the multimodal PFPT treatment, 28 women took part in the interview (Fig. 1). One woman withdrew during treatment because of a serious illness in the family, one woman was lost to follow-up, and one was unavailable to take part in the interview because she was a healthcare provider required to work longer hours because of the coronavirus (COVID-19) pandemic; also, her partner had just been diagnosed with cancer (Fig. 1).

At baseline, the participants’ mean age was 56 (SD 11) years. The women received different oncological treatments: 77% had surgery, 61% had brachytherapy, 48% had external beam radiation therapy, and 52% had chemotherapy. They completed all planned treatments for gynecological malignancies for a median time of 38 (Q1 9 to Q3 70) months before enrolling in the study. Eighteen (58%) women were married, seven (23%) were in a common-law relationship, and six (19%) were single but engaged in a relationship. Three (10%) women reported they had attended a few sessions of multimodal PFPT treatment more than 1 year before their enrollment. Additional details on baseline characteristics can be found elsewhere [11]. During the follow-up period, three women had a cancer recurrence or another cancer and one woman had a severe upper urinary tract infection. No woman stated that she had attempted other treatments for pain or sexual dysfunction after treatment, and only one reported being no longer with her partner. No significant difference in participant characteristics and treatment response was found between those who participated and those who did not participate in the interview.

**Fig. 2:**
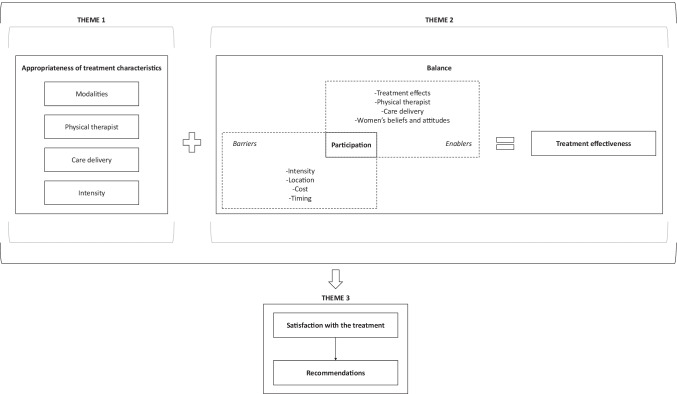
Acceptability of multimodal PFPT treatment

Three themes emerged from the interview transcripts: (1) appropriateness of treatment characteristics; (2) balance between participation and treatment effectiveness; (3) satisfaction with the treatment and recommendations. The themes are described below, and participants’ quotes are presented sequentially according to themes in Tables 1, 2, and 3. Figure 2 illustrates the interactions between the themes and the subthemes.

**Table 1:** Quotes underlying Theme 1

Theme and subthemes	Quote no.	Quotes
1. Appropriateness of treatment characteristics		
1.1. Modalities		
	1	I couldn’t imagine…what a physical therapist was going to do to fix this problem? I didn’t know you could do stretching, and this and that! We think of all the other parts of our body, but the vagina…come on, it doesn’t make any sense! You can’t imagine what the treatment is. Not everyone knows that you have muscles, that you can control them; not everyone has been through this. So, I found it interesting to see and experience this, and it worked. I couldn’t believe it. I was like…“Huh, it can’t be! Could I have done this all those years ago?” I find that extraordinary. (C10)
	2	It [the treatment] was worth it because it put me in touch with the knowledge and the tools that I was unaware of, and that stayed with me. I still have the handout, the exercises, the products...I did not know about moisturizers and lubricants, and you suggested good ones. So, we are now living in a more peaceful period. This is something that remains with me regardless of how demanding it was. I would give a 10 out of 10 for the way it was done, the contact, the information that was given. (C13)
	3	I was impressed! It’s like discovering our body from the inside out. There are areas that we don’t understand or know. How the physical therapist was able to identify a point of resistance with her touch and work it off! I was fascinated and saw the results.…For people who have never done meditation and breathing exercises, hearing: “Take your time to relax and learn to breathe,” it helps too. (C08)
	4	I had muscle tension. My God, it hurt…and I learned with the exercises. I learned how to relax, and it solved my problem. I didn’t think that would happen, so I was very happy....What I liked was the probe. You can really see when you contract and when you release. I found it interesting to visualize because you can try and force it and force it, but…if you don’t do it right, you don’t move forward. (C02)
	5	The exercises with the dilator are the best. It relieves the pressure, the part inside that produces the pain, that hurt.…When the treatment came up and you were using different sizes of dilator, I was happy. If I ever wanted to have a relationship again, the dilator they give [members of the oncology team] would not have worked. It was your number 1 dilator, it’s very tiny.…I liked the progression as I went all the way to being able to use the number 4 dilator comfortably. It made me 10 times more confident because I could get to the size of my husband and I had no fear that I could hurt myself.…The physical therapist was really awesome because she taught me different exercises and that, yes, it’s going be a little painful but if you work through it, the next day, it’s going be easier and it’s going to be easier the next day, etc. Now, I know how to stretch, and I know what pain is okay or not. (C15B)
1.2. Physical therapist	6	[My physical therapist] was a gem. At first, I thought: “How will I be when someone goes in around my private parts?” I didn’t know her and not many people went down there! So, her approach was very important to me. She was…a feather falling on my body; a feather, you don’t feel it. She had…a very humane approach. She was very attentive and showed interest in listening to me. I wasn’t just a number. This touch is very important because we feel alone and talking about it [our sexual problem after cancer] is already difficult. So, having someone so warm and considerate removed all my embarrassment and insecurity. (C17B)
	7	The techniques she [the physical therapist] performed in my vagina could have been excruciating but not with her gentleness and respectfulness. I trusted her. She knew what she was doing.…She did me a lot of good. (C01)
	8	When you start, you don’t really know…It’s intimate, it’s not traditional physical therapy where it’s outside. You must have a great relationship with the therapist…She needs to make us understand.…We usually [my physical therapist and I] talked about how our day was going before getting to the heart of the matter. It was nice because we were like friends. She was very open. She would explain everything to me before doing anything, as if she was preparing me mentally for what was coming. She never came in and…“Bing! Bang! We do it like this!” (C04)
	9	If I had any questions, I could ask them right away. She [the physical therapist] could even guide me if there were things that I was not doing well…There was an exercise that I was not able to do, and she would tell me that it was okay. She was actually able to say to me: “Here’s what we can do.”…I thought the documentation was great and having someone, a physical therapist, to show us exactly how to do it, how to progress, helped a lot. Having subsequent sessions allowed me to validate things. (C14)
	10	I had two physical therapists and found them both welcoming and skilled. The change was easier than I thought because the second physical therapist was aware of where I was, we weren’t starting over.…I was really touched by the quality of their interactions. I had this feeling that they understood me and wanted to understand me. They let me express myself. (C06)
	11	It’s a little embarrassing showing your vulva…I had experienced this during my cancer, it’s not always easy. I had men as gynecologists, but when you go there, you don’t stay an hour, undress and…No, I prefer a woman. It was easier for me. I was more comfortable. I think she can understand more given she is a woman too. (C117)
1.3. Care delivery	12	To be one-on-one with somebody and not have six different people in the room was my main goal. It was the best! It felt private, like she [the physical therapist] was there to care about me, she was there for me.…Gaining the knowledge that I had during the treatment [using telehealth] would not have been as satisfying or efficient. I had contact with the physical therapist. It was more hands-on rather than talking to someone over the phone. I was able to just go and ask her. I was also able to try the exercises and understand how to do the exercises properly without pain. (C15B)
	13	I’ve had a few phone consultations and I don’t feel like we’re getting to the heart of the matter. I need physical contact. At the beginning, she [the physical therapist] was doing all the manual handling, I tried at home and, personally, I was not able to do it…I needed the hands of a professional. I find it [the approach] more personalized. We feel more supported. (C16)
1.4. Intensity	14	The frequency, the number [of sessions] was enough for me. Obviously, there were new things to learn in every session, and it was provided. I found it [the treatment] concentrated, but well concentrated. (C02)
	15	It was quite adequate. If you want to see an improvement, you must do a minimum to learn, to integrate the exercises and the ways of doing things well…to really feel an improvement. (C03)
	16	At the beginning, twelve sessions can be a lot because it is a long time when you consider three months. When you’ve completed it [the treatment] though, you realize that those twelve sessions were worthwhile and necessary to go forward gradually. (C08)

**Table 2:** Quotes underlying Theme 2

Theme and subthemes	Quote no.	Quotes
2. Balance between participation and treatment effectiveness		
	17	I was diligent in my treatments, but it was limiting because there was a lot to be done. At the same time, I don’t think it would have been so effective if we had done less because everything was necessary. The result wouldn’t have been as good.…It was worth it because I saw the results. On the other hand, I feel like my condition has slightly deteriorated since because there have been fewer relationships. I tell myself that the next time we have a relationship, it won’t go far because the pain will be back. Since I did not continue the exercises, I feel I have lost what I had gained during the treatment. That’s a pity. I am very happy to have participated, but today I am a little disappointed that the effects did not stay. (C01)
2.1. Participation and treatment effects		
	18	I’m so glad I participated because it really, really improved my situation. I wouldn’t have thought that there would be so much improvement because I thought the pain was meant to stay. I have no pain anymore, so it really is a miracle. It’s positive. I was lucky to have this treatment and when I think that I could have missed it all!…I would say I started to see differences after the third session. This is when I got more involved as I could say: “OK, it’s true, there is something I can do!” It made me feel good to have positive results. It was encouraging to continue…because at some point you wonder…“Hey! Do I continue or do I stop?” That’s why I kept going. (C09)
	19	The visual provided [by the biofeedback] helped me to see the results of my efforts. It was encouraging too. It was helpful to have it for some exercises. We could really see when I contracted and when I relaxed the muscles. (C03)
	20	It progressed slowly, and it was a good thing. Starting with the smallest [dilator], it is encouraging. When we see that it is possible with a smaller one, we can try with the bigger one and it was like that during the treatment. We went along gradually, when I was ready. (C13)
2.2. Participation and treatment characteristics		
	21	It was a big commitment, and it was winter. It was one more obstacle. It took me like forty-five minutes to go there. At the same time, I told myself: “It’s worth it and I’m going to do it.” So, I went along with it and it ended up very well. What motivated me was the level of knowledge I could acquire to improve the situation.…The physical therapist could have come to my house or we could have done things digitally, but I’m not sure I would have felt very comfortable. We are not necessarily alone at home. You know, husband, children…In my case, I liked getting away to be alone with my physical therapist. I also find that having a direct contact with her [the physical therapist] gives us more confidence. She shows us how to do the exercises. (C115)
	22	It’s $100 per session in the private sector; that’s a lot of money. I couldn’t afford it, a lot of women can’t. This treatment should be free for women who have had cancer. I think the government should pay for it. It seems to me that this is the continuation of the cancer treatment…I saw the benefits and it gives women confidence that there is hope, that you can control the pain. I think it should be offered a few months after the treatments, but every woman is different. It should be offered when the woman feels more comfortable because it’s not easy to let people go there after you’ve been through that [the cancer], you kind of want to put that part of your body away. The treatment could be initiated by giving a leaflet to women and the physical therapist could be available to meet us or call us so that we can communicate our fears, our questions. Then, if we wish and if we are ready, we could go further. (C122)
	23	The treatment gave me the structure I needed. I knew that it was once a week, that I was going to do my exercises, that I was going to have a plan. It gave me the structure of the things I had to do to improve…That’s motivating. I felt like I was well accompanied and doing something that moved me forward. The follow-up really helped me to understand how and why I was doing these things and it was also encouraging because the professional oversees what you are doing, so you engage more. (C08)
	24	During the treatments, [my physical therapist] would explain everything to me, tell me what was going to happen and what to do. There was always a great respect for pain and for privacy, so I was comfortable to go.…When [she] started talking to me about dilators, I thought it was a little weird, but after that it became a game. (*Laughs*). When she showed me different sizes, I named them! (*Laughs*). The first was Brad Pitt. (*Laughs*).…We laughed about it, we had fun.…I really liked [my physical therapist]. The first time my husband and I succeeded in having intercourse, I couldn’t wait to tell her, and she was as excited as I was!…The last day I saw her, it was like I was leaving a friend because I could tell her anything, I would talk to her about anything, even things that I didn’t necessarily tell my husband. She was my confidante!…This treatment gave me an intimate life with my husband, and I thank my physical therapist who made this experience as easy and enjoyable as possible. She was supportive and made me feel comfortable with my own sexuality. She helped me get to know my vagina and the importance of taking care of it. (C10)
2.3. Participation, women’s beliefs and attitudes		
	25	I was ready to do anything to help myself. I thought: “I have to go. What if this can help me?” And I was confident, I was like: “They don’t do this just for fun.” What also helped me were the exercises: it was touching myself, getting to touch what was blocking me. I was no longer saying: “I had cancer, I had treatments, I’m going to stay like this all my life, period.” It’s as if something unblocked and I started to believe that things can get better. So, I engaged more. (C124)
	26	I wanted to see changes, improvement in my life. So, for sure that motivated me and I got involved. When I commit to something, I do it. All my life, I don’t think it ever happened that I gave up midway. Usually when I do something, I do it. I was like: “If there’s anything I can do about it, well I’ll give it a try,” and I enrolled thinking it wouldn’t help much. (C123)
	27	Commitment takes time. If you had told me a year, that might have been something else. But twelve weeks, for me, I would have taken three more at this level. When I commit to something, I commit myself fully.…You must be disciplined. It’s like any treatment, if you do it occasionally, I don’t think the results will be there. It really must be done methodically and regularly. It’s like going to the gym. I called it: “Going to the gym but to my room.” (*Laughs*). I put it in my routine, and it went well. (C04)
	28	I was told it’s twelve, so I said to myself: “I’m doing the twelve,” and on top of that it’s research and it can help other people as well afterwards. It’s true that I was wondering what we were going to do, but I thought: “Let’s try. It must work.” I didn’t think it was going to be so fast and so effective. (C111)
	29	When I learned of the study, I thought to myself: “My God!” I didn’t know what it was, but I was like: “If this can be good, I will go and see, out of curiosity, and if it doesn’t help, I will stop going.” The more I went there, the more it gave me something. The more I saw that it wasn’t that bad, I just kept going and at one point I said: “OK, I’m going until the end.” I saw all the improvement and all the things that I could do by myself. In the end, it didn’t matter if it was twelve or fourteen sessions; it didn’t bother me. Basically, I was aiming for the result, and I was ready to give it my all. (C100)
	30	He [my partner] had already taken several leaves to come with me to the hospital, so he couldn’t come to the sessions, but he knew…I talked about it a lot and he had seen what the exercises were because at the beginning I was having trouble with the dilator, doing the exercises myself, and he tried to help me. Anyway, he knew what it was all about, and he supported me, he was understanding. (C18)

**Table 3:** Quotes underlying Theme 3

Theme	Quote no.	Quotes
3. Satisfaction with the treatment and recommendations		
	31	The attitude was very courteous and warm; I was really happy with the approach. Everything was top-notch, the number of sessions versus the results you want to achieve. I felt that everything had been calculated correctly to allow progress and results. If it had been for a shorter period, it wouldn’t have been complete. The treatment provided enough results, enough to say…“OK, I am satisfied, it worked.”…Physical therapy should be part of survivorship care.…Breast reconstruction after breast cancer is covered by the hospital but costs related to “perineal reconstruction” are not. Breast reconstruction is necessary for the woman, for her life, for her to continue to live in harmony, for her vision of herself, for her esteem; yes, but “perineal reconstruction” is just as important. It’s even dangerous if nothing is done: the vagina may shrink, close up…the pain…it’s a need. This is clearly a need, but it is not automatically offered after gynecological cancer as with breast cancer. So, there is something that is not fair. (C12)
	32	The treatment met my needs, maybe more, I didn’t think I was going to appreciate it that much.…It brought me more than I thought. I didn’t think it was going to bring me so much.…It exceeded my expectations, so I’m very happy with all the treatment. And if I happen to have other problems, I think I will consult the same physical therapist because I liked her.…Ah! I am very satisfied, very, very. I recommend it. Specialists should talk to us about it. It’s up to us to decide whether we want it or not. It would be perfect if they could tell us…“If you want to go to physio, you can go, it’ll make you a lot better,” but nobody talks about it. I don’t understand, we should be told. We should be offered this treatment. I would never have thought that there were physical therapy treatments for that. They do it for any other surgery, why not for that? (C117)
	33	I had met a sex therapist at the hospital and had maybe three appointments. She gave me some information to order a dilator, but you know, I didn’t. I had to get the dilator. There was a document I didn’t understand, and I didn’t take it seriously. I was like on my own and gave up. She was giving me a recipe. I had to go, buy and follow the recipe by myself. I also felt that the sex therapist was working more on my relationship issues rather than my vagina which was painful after the surgery…So, I appreciated my experience in the study much more. If the sex therapist had referred me to physical therapy, I would have understood better. Any woman who has surgery should have the opportunity to have this treatment; when we are followed up for cancer recurrence, that should be part of the treatment automatically as well, a convalescence. (C103)
	34	In a 1000-piece puzzle, when a piece is missing, it won’t work. Physical therapists, as much as nurses, beneficiary attendants, doctors, etc., are essential in the healthcare network. I can see that this treatment was essential for me. I would have been willing to pay for it but not everyone can afford it. So, I think, in the same sense as someone with back pain, a bad knee or anything else, these physical therapy treatments are essential. It should be offered to everyone. (C123)

### Theme 1: Appropriateness of treatment characteristics

#### Subtheme 1.1: Modalities

Almost all participants did not know about multimodal PFPT at the beginning of the study, which led them to believe that this treatment would not alleviate dyspareunia or sexual dysfunction (quote 1). However, all of them acknowledged at some point that, while gaining knowledge, this treatment made sense and was suitable for improving sexual and pelvic health (quote 1). Our cohort did not express a preference regarding the modalities as all were perceived as helpful and complementary (i.e., the educational module, manual therapy techniques, pelvic floor muscle exercises with biofeedback, and home exercises including insertion exercises with a dilator), and women underlined that the treatment provided them with useful knowledge and tools that lasted over time (quotes 2 to 5).

#### Subtheme 1.2: Physical therapist

All women expressed their appreciation of their physical therapist (quotes 6 to 11). They described this appreciation by detailing their therapist’s great humane qualities (e.g., considerate, empathic, gentle, kind, and respectful), competency, and skills (quotes 6 to 10). These features were perceived as essential to help women confront, manage, and reduce their sexual problems (quotes 6 to 10). The physical therapist was viewed as an invaluable asset as the participants emphasized how she set the pace, led the treatment in a sequential and predictable manner, was available for women to discuss any issues, and adjusted the modalities from session to session (quotes 8 to 10). It should be noted that those who had more than one treating physical therapist reported they were comfortable because they did not feel their treatment was jeopardized (quote 10). While there was no treating male physical therapist in the current study, several participants stressed their preference for being treated by a woman (quote 11).

#### Subtheme 1.3: Care delivery

All participants reported that they appreciated the treatment being offered individually and in person (quotes 12 and 13). Several women specified that they would not have been comfortable to participate in a group intervention to discuss the intimate topic of pain and sexuality after cancer (quote 12). Participants felt that the physical contact with the physical therapist provided them with personalized advice and feedback (quotes 12 and 13). Participants concurred that it allowed them to benefit from the techniques performed by the therapist, which would have been difficult in a group or telehealth intervention (quotes 12 and 13).

#### Subtheme 1.4: Intensity

Treatment intensity was depicted in terms of number and frequency of sessions and home exercises. Most participants found the number of sessions (i.e., 12) and the frequency (i.e., one session per week and home exercises five times per week) appropriate for learning and for noticing important effects (quotes 14 and 15). A few women described the treatment as demanding at first (quote 16). Nonetheless, all of them acknowledged over time that this intensity was relevant (quotes 15 and 16).

### Theme 2: Balance between participation and treatment effectiveness

According to the quotes of Subtheme 1.4, women designated the multimodal PFPT treatment as acceptable by weighing their efforts (i.e., participation) against the results they obtained (i.e., treatment effectiveness) (quote 17). As our cohort attributed importance to this ratio, participants described the enablers that overcame the barriers of women’s participation in the multimodal PFPT treatment. Participation was portrayed as the level to which they conformed to the treatment as prescribed (i.e., attendance at sessions and adherence to home exercises) and followed the advice given by the treating physical therapist. The enablers and barriers of participation related to treatment effects, treatment characteristics, and women’s beliefs and attitudes are presented below.

#### Subtheme 2.1: Participation and treatment effects

A large proportion of our cohort reported experiencing significant positive effects [11–13] after the multimodal PFPT treatment, some specifying that these began to appear as early as the third session (quote 18). As women were also able to observe their progress (e.g., increase in muscle control with biofeedback or upgrading the size of dilator) during the treatment (quotes 19 and 20), they explained that they were encouraged to pursue their efforts which, in turn, increased the treatment effectiveness (quote 18). It should be pointed out that the majority of our cohort reported that the treatment effects were maintained during the follow-up period while a few admitted that these were attenuated over time [14].

#### Subtheme 2.2: Participation and treatment characteristics

As deduced from quotes of Subtheme 1.4 and Subtheme 2.1, the high treatment intensity was cited as the most important factor that could discourage women from participating in the multimodal PFPT treatment (quote 21). Although these factors were not as prominent or relevant in the current study, the location, the cost, and the timing of the treatment were raised by some women to potentially impede participation (quotes 21 and 22). This led women to make a few suggestions to adjust the treatment. They suggested that initiating the treatment through other types of care delivery, giving first-hand information to manage sexual problems and subsequently offer more intensive care could be considered, particularly when environmental barriers prevent women from attending the treatment (i.e., women living in remote areas or when transportation is unsafe due to the weather) (quotes 21 and 22). As reflected in the quotes from Subtheme 1.3, participants implied that care delivery facilitated their participation in the multimodal PFPT treatment. Moreover, several of them reported that the structure and the supervision provided, with the back-and-forth with their therapist, motivated them to conform to the treatment (quotes 23 and 24). The physical therapist, through her positive and supportive attitude, was largely reported as a facilitator for women’s participation, and many women emphasized they enjoyed being with their therapist and how their relationship made them more committed to the treatment (quote 24).

#### Subtheme 2.3: Participation, women’s beliefs and attitudes

Although most of the participants did not know what multimodal PFPT entailed (Subtheme 1.1), the women stated that they were prepared and even determined to participate and complete the treatment (quotes 25 to 29). They explained how their needs and goals (i.e., willingness to improve their situation or attempt to reach the highest effectiveness), their beliefs regarding their sexual problems or engagement in treatment, their personality trait (i.e., highly committed person), and the research context (i.e., opportunity to help other women) played a role in their participation (quotes 25 to 29). Treatment expectations were diversified in our cohort (i.e., no expectations to high expectations) but were not perceived by women as a determinant of participation (quotes 26 and 28). Some women described how much their beliefs and attitudes changed during the treatment (quotes 25 and 29), and some said that their partner contributed to their participation (quote 30).

### Theme 3: Satisfaction with the treatment and recommendations

Following the logical extension of Theme 1 and Theme 2, the participants said they were highly satisfied as they explained their positive experiences during the multimodal PFPT treatment and the balance between their participation and the treatment effectiveness they perceived (quotes 31 and 32). They particularly expressed their satisfaction when the treatment outcomes met their needs or reached, or even exceeded, their initial expectations (quote 32). To emphasize their satisfaction, some women compared their experience of multimodal PFPT with previous unsatisfactory treatment attempts (quote 33). Consequently, all participants recommended multimodal PFPT for women who have been treated for gynecological malignancies (quotes 31 to 34). Our cohort also stressed that multimodal PFPT should be automatically offered, free of charge, in the gynecological cancer care continuum, particularly considering that physical therapy services are supplied to treat other outpatient populations (e.g., after breast cancer treatment or orthopedic surgery and pain conditions) (quotes 31 to 34). Moreover, several participants highlighted the complementary role of physical therapists in multidisciplinary survivorship care (quotes 33 and 34).

## Discussion

This is the first qualitative study to examine extensively the acceptability of multimodal PFPT. This treatment was found acceptable according to women who developed dyspareunia after gynecological malignancies. Our cohort described how the treatment was appropriate in terms of modalities, physical therapist, care delivery, and intensity. While the treatment intensity could be viewed as demanding, all participants stressed that it was relevant to see significant improvements. They explained that noticing the effects during the treatment encouraged them to pursue their participation. The physical therapist and the care delivery (i.e., treatment-related factors) as well as the women’s beliefs and attitudes (i.e., women-related factors) were also identified by women to facilitate their participation. Participants expressed their high satisfaction with the treatment as they detailed their positive experiences and the balance between their participation and the treatment effectiveness they perceived. All women recommended this multimodal PFPT treatment.

The multimodal PFPT treatment was found acceptable as our cohort described the modalities, the physical therapist, the care delivery, and the intensity as appropriate. Very few studies have examined the acceptability of multimodal PFPT in similar terms in gynecological cancer survivors [21–23]. The study of Lindgren et al. [23] described gynecological cancer survivors’ views and experiences (*n* = 13) of pelvic floor muscle training for treating incontinence. Although women had little or no experience with pelvic floor muscle training, they had a positive attitude toward this treatment [23], which is in line with the input of our cohort who did participate in a multimodal PFPT treatment. Women from the study of Lindgren et al. [23] also underlined the importance of being instructed by a competent professional [23], which further emphasizes the role of the physical therapist, as highlighted in our study. Data available also imply that the professional’s supportive role and dilator use are helpful for resuming sexual activity [21, 22], which is consistent with our study. In contrast to studies supporting internet- and group-based interventions for sexual and psychosocial problems [24, 25], our participants expressed that they preferred an individual and in-person treatment approach. Women explained how it allowed them to receive relevant information and benefit from manual therapy techniques and proper feedback (e.g., guidance). These results stress the importance of designing and offering treatments according to women’s condition (e.g., dyspareunia). Given that our cohort perceived the modalities as helpful and complementary and did not express preference for specific modalities, all of them can be made available to women.

The participants of the present study compared their participation and the treatment effectiveness they perceived and deemed the multimodal PFPT treatment as acceptable. While the intensity of the treatment could have burdened some women, it was reported as important to see significant improvements. This is the first qualitative study showing how treatment-related and women-related factors can counterbalance the burden of a treatment. Several participation barriers (e.g., financial constraints, perceived lack of utility, time constraints, and travel issues) and enablers (e.g., increased knowledge, gain in tools and skills, perceived improved well-being, sense of validation and support, and enhanced sense of empowerment) [26, 27] of PFPT treatments have been reported in women with dyspareunia but without a history of cancer, which are in line with those identified in the current study. Furthermore, women reported that participation is a key element to reaching the highest treatment effectiveness. Although no interventional study has examined the participation behavior of gynecological cancer survivors with sexual problems in treatments, studies conducted in women with no history of cancer presenting with pelvic floor disorders highlighted that participation is important in the context of PFPT [28, 29]. Given the attributed importance of participation behavior in treatment effectiveness, future studies should include measures of participation behavior. Further work should also compare the effects of different types of care delivery and level of supervision on participation behavior and treatment effectiveness to determine which should be emphasized.

Our participants described their high satisfaction according to their positive experiences with the multimodal PFPT treatment characteristics and the balance between participation and treatment effectiveness. Moreover, all participants recommended the treatment, and some even suggested slight adjustments to make it more accessible to women. Consequently, results support the implementation of the multimodal PFPT treatment in the gynecological cancer care continuum. Our findings also support and may refine the theoretical framework of acceptability [17]. It has been hypothesized that acceptability (i.e., appropriateness of treatment based on cognitive and emotional responses) likely influences participation behavior with the treatment [17], which has been shown in the current study. Our results also suggest that satisfaction is interlaced with the appropriateness of treatment in a dynamic framework of acceptability in which different factors influence participation behavior. For instance, women said that they were satisfied as they perceived beneficial effects because of their participation in the treatment. This further encouraged their participation and, ultimately, counterbalanced the burden induced by the regimen intensity. This reflects the interaction between the multiple facets of treatment acceptability [30], which should be considered comprehensively.

Some limitations should be considered when interpreting the findings of this study. The generalizability is limited by the sampling method. Our sample was composed of women who agreed to participate in the multimodal PFPT treatment. This sample, however, allowed us to understand treatment acceptability, including the dynamic interplay of factors influencing participation which is essential in the context of rehabilitation [28, 29]. The participants were mostly Caucasian (97%), had a stable sexual partner, and were willing to resume sexual activities with vaginal penetration. Our results can be generalized to women who have similar characteristics. Although women’s age, cancer stage diagnosis, cancer treatments, time elapsed since these treatments, level of education, and annual income varied in our cohort [11], these characteristics did not appear to significantly influence the acceptability of the multimodal PFPT treatment. Another strength of this study is the semi-structured guide, which was constructed based on a well-known framework of acceptability [17]. The in-depth qualitative interviewing also deepened our understanding of the treatment acceptability. The interviewer was not involved in the treatment of any participant, limiting the social desirability bias. Different methods were also used to reduce the researchers’ subjectivity in the data interpretation. Interviews were recorded, transcribed, and analyzed using an iterative and inductive approach. This allowed the emergence of innovative and contextual themes. Data saturation during data collection was reached, followed by inductive thematic saturation during analysis.

In conclusion, multimodal PFPT was found acceptable by gynecological cancer survivors. Findings provide a deeper understanding of this treatment’s acceptability which involves the appropriateness of its characteristics, the balance between participation and effectiveness, and satisfaction. Multimodal PFPT can be implemented in follow-up care in gynecological oncology. Selecting the most appropriate modalities, therapist, care delivery, and intensity is a critical step for implementation.

## Supplementary information

ESM 1 (DOCX 27.5 kb)
