# Supplementary material for: Acceptability of multimodal pelvic floor physical therapy to treat dyspareunia after gynecological malignancies: a qualitative study of women’s views and experiences
Source: Int Urogynecol J. 2022 Aug 10;34(5):1061–73. doi: 10.1007/s00192-022-05304-4 (PMC9364276; doi:10.1007/s00192-022-05304-4)
Supplement: Supplementary file 1 — (DOCX 27.5 kb) [file 192_2022_5304_MOESM1_ESM.docx]

**Supplementary material. Semi-structured interview guide [1].**

French version

Questions générales

1. Décrivez-moi vos sentiments et vos pensées à l’égard du traitement multimodal de physiothérapie que vous avez reçu.

a. Quels sont vos pensées et sentiments concernant…

- le nombre de séances?

- le contenu des séances?

- la physiothérapeute?

- le déroulement du traitement?

- l’efficacité du traitement?

- votre participation?

b. Est-ce que ces aspects sont appropriés ou inappropriés?

Déclencheurs : Expliquez comment c’est approprié. Expliquez comment c’est inapproprié.

c. Qu’avez-vous trouvé le plus ou le moins utile dans le traitement?

d. Qu’avez-vous le plus ou le moins aimé du traitement?

2. Parlez-moi plus de votre participation au traitement.

a. Qu’est-ce qui a motivé ou aurait pu motiver votre participation?

b. Qu’est-ce qui a entravé ou aurait pu entraver votre participation?

c. Y a-t-il d’autres facteurs que nous devrions prendre en considération si nous entreprenons d’offrir ce traitement à d’autres femmes?

3. Comment évalueriez-vous votre satisfaction à l’égard du traitement que vous avez reçu?

Déclencheurs : Qu’est-ce qui définit votre satisfaction?

4. Quelles sont vos suggestions pour améliorer le traitement?

Autres exemples de déclencheurs qui ont été utilisés pour obtenir plus d’information sur la perspective et l’expérience des participantes à l’égard de l’acceptabilité du traitement multimodal de physiothérapie :

1. Parlez-moi plus de [xxx].

2. Que voulez-vous dire lorsque vous dites [xxx]?

3. Décrivez-moi comment le traitement a affecté votre participation.

4. Décrivez-moi comment votre participation a influencé l’efficacité du traitement.

5. Expliquez-moi ce qui vous incite à recommander ce traitement à d’autres femmes.

English version

General questions

1. Describe to me your thoughts and feelings about the multimodal pelvic floor physical therapy treatment that you received.

a. What are your thoughts and what are your feelings about…

- the number of sessions?

- the content?

- the physical therapist?

- the course of the treatment?

- the effectiveness of the treatment?

- your participation?

b. Are these aspects of the treatment appropriate or not appropriate?

Probes: Explain how it is appropriate. Explain how it is not appropriate.

c. What did you find the most or the least useful in the treatment?

d. What have you appreciated the most or the least of the treatment?

2. Tell me more about your participation in the treatment.

a. What motivated or could have motivated your participation?

b. What impeded or could have impeded your participation?

c. Are there any other factors that we should consider if we plan to offer this treatment to other women?

3. How would you rate your satisfaction regarding the treatment you received?

Probes: What defines your satisfaction?

4. What are your suggestions to improve the treatment?

Other examples of probes to obtain in-depth information about participants’ views and experiences regarding the acceptability of multimodal PFPT treatment:

1. Tell me more about [xxx].

2. What do you mean when you say [xxx]?

3. Describe to me how the treatment affected your participation.

4. Describe to me how your participation influenced the effectiveness of the treatment.

5. Explain to me what makes you recommend this treatment to other women.

[1] Cyr MP et al. Acceptability of multimodal pelvic floor physical therapy to treat dyspareunia after gynecological malignancies: a qualitative study of women’s views and experiences. Int Urogynecol J. 2022.
